# Basal Forebrain Cholinergic Innervation Induces Depression-Like Behaviors Through Ventral Subiculum Hyperactivation

**DOI:** 10.1007/s12264-022-00962-2

**Published:** 2022-11-07

**Authors:** Nana Yu, Huina Song, Guangpin Chu, Xu Zhan, Bo Liu, Yangling Mu, Jian-Zhi Wang, Yisheng Lu

**Affiliations:** 1 Department of Physiology, School of Basic Medicine, Tongji Medical College, Huazhong University of Science and Technology, Wuhan, 430030 China; 2 Department of Pathophysiology, Key Laboratory of Ministry of Education for Neurological Disorders, School of Basic Medicine, Tongji Medical College, Huazhong University of Science and Technology, Wuhan, 430030 China; 3 Department of Physiology and Pathophysiology, Sino-UK Joint Laboratory of Brain Function and Injury of Henan Province, School of Basic Medicine, Xinxiang Medical University, Xinxiang, 453003 China; 4 Department of Neurobiology, School of Basic Medicine, Huazhong University of Science and Technology, Wuhan, 430030 China; 5 Department of Otorhinolaryngology, Union Hospital, Tongji Medical College, Huazhong University of Science and Technology, Wuhan, 430022 China; 6 Institute of Brain Research, Collaborative Innovation Center for Brain Science, Huazhong University of Science and Technology, Wuhan, 430030 China

**Keywords:** Depression, Medial septum, Subiculum, Acetylcholine, Circuit

## Abstract

Malfunction of the ventral subiculum (vSub), the main subregion controlling the output connections from the hippocampus, is associated with major depressive disorder (MDD). Although the vSub receives cholinergic innervation from the medial septum and diagonal band of Broca (MSDB), whether and how the MSDB-to-vSub cholinergic circuit is involved in MDD is elusive. Here, we found that chronic unpredictable mild stress (CUMS) induced depression-like behaviors with hyperactivation of vSub neurons, measured by c-fos staining and whole-cell patch-clamp recording. By retrograde and anterograde tracing, we confirmed the dense MSDB cholinergic innervation of the vSub. In addition, transient restraint stress in CUMS increased the level of ACh in the vSub. Furthermore, chemogenetic stimulation of this MSDB-vSub innervation in ChAT*-*Cre mice induced hyperactivation of vSub pyramidal neurons along with depression-like behaviors; and local infusion of atropine, a muscarinic receptor antagonist, into the vSub attenuated the depression-like behaviors induced by chemogenetic stimulation of this pathway and CUMS. Together, these findings suggest that activating the MSDB-vSub cholinergic pathway induces hyperactivation of vSub pyramidal neurons and depression-like behaviors, revealing a novel circuit underlying vSub pyramidal neuronal hyperactivation and its associated depression.

**Supplementary Information:**

The online version contains supplementary material available at 10.1007/s12264-022-00962-2.

## Introduction

Major depressive disorder (MDD) is one of the most severe and common mental disorders characterized by persistent sadness and emotional numbness, usually comorbid with cognitive dysfunction and physical symptoms, and affects >10% of the worldwide population during their lifetime [1]. However, ~30% of MDD patients are resistant to the current first*-*line antidepressant treatments, including serotonin and norepinephrine reuptake inhibitors or modulators [2–4]. Despite enormous efforts, the neurobiological mechanisms underlying MDD are still largely unknown [5, 6], hampering the development of novel treatments for drug-resistant patients.

According to the structural neuroimaging and postmortem studies, the frontal lobe and hippocampus are the most common regions manifesting a total volume decrease in MDD patients [7, 8]. In addition, the volume decrease of the hippocampus in MDD patients can be reversed by antidepressant or electroconvulsive treatments [9, 10], suggesting the importance of the hippocampus in MDD. The ventral hippocampus is more responsible for emotional information processing than the dorsal hippocampus, connecting to other brain regions that are dysfunctional in MDD, including the prefrontal cortex [11], striatum [12], and amygdala [13]. The dentate gyrus and subiculum are the main subregions that control the input and output connections of the hippocampus, respectively, and these subregions are both affected in MDD patients [14, 15]. According to recent studies, the dentate gyrus plays a vital role in depression [16]; however, much less is known about the mechanisms of action of the subiculum in the brain network of MDD patients.

The cell bodies of cholinergic neurons in the brain are mainly in the basal forebrain and brainstem, and accumulating evidence suggests that the cholinergic system in the basal forebrain is critical in mood regulation [17]. Both human and animal studies have shown that acetylcholine (ACh) is increased in the brain during MDD [18–22]. Differing from the nucleus basalis magnocellularis in the forebrain targeting the cortex, the cholinergic neurons in the medial septum and diagonal band of Broca (MSDB) densely project to the hippocampus; acetylcholinesterase inhibitors in the hippocampus induce depression-like symptoms [21]; and the muscarinic ACh receptor antagonist scopolamine has rapid*-*onset and sustained antidepressant effects in depressed patients [23]. However, the effect of ACh on the hippocampus is still controversial, depending on the timing of cholinergic input [24] and the stimulation frequency [25].

Here, we assessed the activation of ventral subiculum (vSub) pyramidal neurons in the mouse depression model of chronic unpredictable mild stress (CUMS). Chemogenetic activation of MSDB cholinergic neurons activated pyramidal neurons in the vSub and induced depression-like behaviors. A muscarinic ACh receptor antagonist in the vSub reversed the depression induced by CUMS and dysfunction of the MSDB-vSub circuit. These results support an essential role of the cholinergic modulation of depression in the MSDB-vSub circuit.

## Materials and Methods

### Animals

ChAT*-*Cre (stock No: 006410) and GAD2-Cre (stock No: 028867) mice were purchased from the Jackson Laboratory. All mice were group-housed at a stable temperature (23°C–25°C), exposed to a 12-h light/dark cycle with food and water provided *ad libitum* unless otherwise indicated. All experiments were approved by the Animal Care and Use Committee of Huazhong University of Science and Technology guidelines. All efforts were made to minimize suffering and the number of animals used.

### Procedure for Chronic Unpredictable Mild Stress (CUMS)

Mice were singly housed before the CUMS protocol started. Briefly, this protocol consisted of four long-term stressors (for 24 h) (cage tilting, food or water deprivation, and placement in an empty cage), and seven short*-*term stressors: cold (4℃ for 1 h), white noise (1h), heat (50℃), tail pinch (2 min), cage shaking (30 min), restraint (4 h), and pepper smell (4 h). Each session consisted of one long-term stressor and two short*-*term stressors were applied randomly, with a break of at least 2 h between them. One session was applied per day for 14 days.

### Behavioral Testing

For all behavioral tests, mice were handled in the testing rooms for at least 40 min per day for three days before taking behavioral measurements. All behavioral tests were applied 24 h apart, and all instruments were from Shanghai XinRuan Information Technology Co., Ltd. Mice were returned to their home cage after each session.

### Sucrose Preference Test (SPT)

Mice were singly housed and acclimatized with two bottles of 1% sucrose or regular water for 24 h, and the positions of the bottles were switched every 12 h. After water deprivation for 24 h, sucrose and water intake were measured in a 2-h test. Sucrose preference was reported as (Consumed sucrose) / (Consumed sucrose + Consumed water) × 100%.

### Forced Swim Test (FST)

The mice were gently placed in a glass cylinder (30 cm high, 10 cm in diameter) filled with 20 cm deep 23°C–25°C water. Total immobile time was scored by analysis of videotapes of the last 5 min in the 6-min test.

### Tail Suspension Test (TST)

Mice were gently suspended ~50 cm above a table by the tail with adhesive tape attached to a hook, and the behavior was videotaped. The immobile time was scored by analysis of videotapes for the last 5 min in the 6-min test. Mice that climbed up their tails were excluded.

### Open Field Test (OFT)

The mice were gently placed at the center of a rectangular chamber (50 cm × 50 cm × 50 cm^3^), and movement was monitored for 5 min using an automated video tracking system (Supermaze 2.0, Softmaze, China). After each trial, the apparatus was swabbed with 75% alcohol to eliminate olfactory cues. The distance traveled during the session was measured.

### Virus Injection

Mice were anesthetized with 1% pentobarbital sodium (35 mg/kg, i.p.) and placed in a stereotaxic frame (RWD Life Science). Viruses were injected using a glass micropipette connected to a 10-µL NanoFil syringe. The infusion rate was 80 nL/min controlled by an UltraMicroPump with a Micro4 controller (World Precision Instruments). At the end of injection, the micropipette remained in place for 10 min before withdrawal.

AAV-Ef1α-DIO-hM3Dq-eYFP or AAV-Ef1α-DIO-eYFP from BrainVTA (Wuhan, China, 600 nL, 3–8×10^12^ vg/mL) was infused into the MSDB using the coordinates (in mm from bregma): A/P +0.98; M/L 0; D/V −4.8, −4.5, and −4.2. The mice were subjected to behavioral tests 4 weeks after virus injection. Brains were then sectioned to verify the infusion sites. Data were excluded if virus expression was misdirected.

### Immunostaining

Mice were anesthetized with 1% pentobarbital sodium and perfused transcardially with 0.1 mol/L PBS followed by 4% paraformaldehyde (PFA) in 0.1 mol/L PBS. The brain was removed and post-fixed overnight in 4% PFA at 4℃, then equilibrated in 30% sucrose. After embedding in OCT, the brain tissue was cut at 30 µm on a Leica cryostat or cooled-stage microtome and stored in 0.1 mol/L PBS at 4℃. For immunostaining, sections were incubated with 0.5% Triton X-100 (PBST) for 30 min at room temperature and 5% BSA in PBST for 30 min at room temperature, then incubated in primary antibodies (ChAT 1:100, Millipore, AB144P, c-fos 1:300, Abcam, ab214672; CaMKIIα 1:300, GeneTex, GTX127939; GAD67 1:300, Millipore, MAB5406) in 5% BSA in PBST overnight at 4℃. After washing 3 times with PBST, sections were incubated in fluorophore-conjugated secondary antibodies (1:500, Invitrogen) for 1 h at 37℃. After another 3 washes in PBST, the sections were incubated with DAPI (Beyotime Biotechnology) and mounted on glass slides with a fluorescence mounting medium. Fluorescent signals were imaged with a confocal laser scanning microscope (LSM 780, Zeiss, Germany). The number of fluorescent cells was counted by ImageJ 1.48v (National Institutes of Health, USA).

### Retrograde Tracing with Cholera Toxin Subunit B (CTB)

Alexa Fluor-555-conjugated CTB (1 μg/μL, 300 nL, Invitrogen) was bilaterally injected into the vSub (A/P −4.1; M/L ±3.5; D/V −3.5 mm relative to bregma) of C57BL/6J mice to retrogradely label neurons projecting to the vSub. After 10 days–12 days, the mice were sacrificed, and the expression of CTB in the MSDB was detected with a confocal laser scanning microscope (LSM 780, Zeiss, Germany).

### Fiber Photometry Recording

Mice were anesthetized with 1% pentobarbital sodium, and AAV9-hSyn-GACh2.0 (1.5 × 10^12^ vg/mL, 300 nL) was unilaterally injected into the vSub (A/P −4.1; M/L −3.5; D/V −3.5 mm relative to bregma). After 3 weeks, mice were anesthetized again, and a mono optical fiber (200 μm outer diameter, 0.37 numerical aperture) was unilaterally inserted close to the vSub (A/P −4.1; M/L −3.5; D/V −3.2 mm relative to bregma) and fixed to the skull with glass ionomer cement. After 1 week of recovery in the home cage and 14 days of CUMS treatment, mice were subjected to the fluorescent signals test. For the fiber photometry recording, mice were gently held in the experimenter's left hand for 5s to imitate transient stress. Fluorescent signals of GACh were stimulated (405 nm) and recorded by an optical fiber recording system (Thinker Tech, Nanjing Biotech, China). The signals before (F0) and during stress (F) were recorded, and the changes in fluorescence were calculated as (F – F0)/F0.

### *In Vitro* Electrophysiology

*In vitro* electrophysiological recordings were made as previously described [26]. The mice were anesthetized with 1% pentobarbital sodium, and the brains were quickly removed and placed in chilled ice-cold modified artificial cerebrospinal fluid (ACSF) containing (in mmol/L): 110 choline chloride, 2.5 KCl, 1.3 NaH_2_PO_4_, 25.0 NaHCO_3_, 0.5 CaCl_2_, 7 MgCl_2_, 20 glucose, 1.3 Na-ascorbate, 0.6 Na-pyruvate. Coronal brain slices (300 μm) were cut on a vibratome (VT1000S, Leica, Germany) and transferred to a holding chamber containing oxygenated regular ACSF, composed of (in mmol/L) 125 NaCl, 2.5 KCl, 1.3 NaH_2_PO_4_, 25 NaHCO_3_, 2 CaCl_2_, 1.3 MgCl_2_, 1.3 Na-ascorbate, 0.6 Na-pyruvate, 10 glucose, incubated at 34.5℃ for 30 min and at 25 ± 1℃ for an additional 1 h. All solutions were saturated with 95% O_2_ / 5% CO_2_ (vol/vol). For recordings, slices were held in a small chamber superfused with oxygenated ACSF at room temperature (2 mL/min). Cells were visualized under an upright microscope (Olympus, BX51WI) with infrared differential interference contrast optics.

To characterize the E/I ratio, monosynaptic eEPSCs and eIPSCs were recorded in vSub pyramidal neurons by patch-clamp in the whole-cell configuration. Pyramidal neurons were identified by their pyramidal shape and spike frequency adaptation induced by prolonged depolarizing current injection. A concentric bipolar stimulating electrode (World Precision Instruments) was positioned on the synaptic input path in the vSub, ~200 μm from the recording electrode. Recording microelectrodes (3 MΩ–5 MΩ) were filled with an intracellular recording solution containing (in mmol/L): 125 Cs-methanesulfonate, 5 CsCl, 10 Hepes, 0.2 EGTA, 1 MgCl_2_, 4 Mg-ATP, 0.3 Na_2_-GTP, 10 Na_2_ phosphocreatine, and 5 QX314 (pH 7.2, 290 mOsm–300 mOsm). eEPSCs and eIPSCs were recorded at the reversal potential of the glutamate receptor (−70 mV) or the GABA_A_ receptor (0 mV).

Action potentials were evoked by a series of 400-ms current injections from 0 pA to 400 pA in 50-pA steps. A high-K^+^ intracellular solution filled the recording pipette (4 MΩ–6 MΩ) containing (in mmol/L): 140 potassium-gluconate, 5 KCl, 2 MgCl_2_, 0.2 EGTA, 10 HEPES, 4 Mg-ATP, 0.3 Na_2_-GTP, 10 Na_2_ phosphocreatine (pH 7.2, 290 mOsm–300 mOsm).

To verify chemogenetic activation or inhibition, action currents of MSDB cholinergic neurons labeled with eYFP were recorded in the cell-attached voltage-clamp configuration at 0 mV. Cell-attached current recordings were made using pipettes filled with ACSF (4 MΩ–6 MΩ). All data were recorded with an Axonpatch 700B amplifier (Molecular Devices) and a Digidata 1550B A-to-D converter (Molecular Devices) at 10 kHz, low-pass filtered at 2 kHz, and analyzed using ClampFit 10.2 software (Molecular Devices).

### *In Vivo* Electrophysiology

Mice were anesthetized with 20% urethane (0.15 mL/20 g, i.p.) and mounted on a stereotaxic frame. To record extracellular spikes from the MSDB (A/P: +0.98; M/L 0; D/V −4.0 mm relative to bregma) or the vSub (A/P −4.1; M/L −3.5; D/V −3.5 mm relative to bregma), four tetrodes of four twisted Formvar-coated platinum-iridium probes (17 μm; California Fine Wire) were attached to a custom microdrive with Epoxy (Precision Fiber Products). The assembled microdrive was secured to the skull by four anchor screws and dental cement, with the tetrodes targeted at the appropriate stereotaxic coordinates. After the mice recovered from the surgery (~1 week after implantation), the microdrive was advanced gradually to lower the tetrodes to the desired anatomical location. All electrophysiological recordings were made using the OmniPlex D Neural Data Acquisition System (Plexon Inc.). The electrical signal was filtered at 0.05 Hz–8,000 Hz, amplified at a gain of 250–5,000, and digitized at 40 kHz. Spikes were sorted with Off-Line Sorter software (Plexon Inc.) using automatic sorting methods and manual checking of single-unit isolation. Putative MSDB cholinergic and vSub pyramidal neurons were identified according to the reported criteria [27]. To deliver drugs, a cannula (100 µm silica capillary tubing; Polymicro Technologies) was attached to the tetrodes with the tip extending 300 µm beyond the tetrodes. Thirty minutes after Clozapine N-oxide (CNO) injection (3 mg/kg i.p., MedChemExpress, HY-17366), atropine (2.5 μg, 400 nL per side, Sigma, A0257) or mecamylamine (0.5 μg, 300 nL per side, Sigma, M9020) were microinjected (100 nL/min) into the vSub through the cannula. Ten minutes later, the data were collected. After *in vivo* recordings and behavioral experiments, mice were anesthetized with 1% pentobarbital sodium and then perfused with 4% PFA. Their brains were sectioned to verify the electrode and cannula placement. Mice were excluded if the implantation site was incorrectly positioned.

### *In Vivo* Pharmacological Approach

Mice were anesthetized with urethane for *in vivo* electrophysiological recording or isoflurane for behavioral tests, then mounted on a stereotaxic frame for cannula implantation, Two 26-gauge guide cannulas (RWD Life Science, 62004) were implanted in the vSub (A/P −4.1; M/L ±3.5; D/V −3.5 mm from bregma) on both sides of the brain, and secured to the skull with two anchor screws and dental cement. For microinfusion, a 33-gauge internal cannula (RWD Life Science, 62204) connected to a 5-µL syringe (Hamilton) was inserted into the guide cannula. CNO (1 mmol/L, 300 nL per side), atropine (2.5 µg, 400 nL per side), or muscimol (0.1 mmol/L, 300 nL per side, Sigma, G019) at 100 nL/min were infused into the vSub on both sides controlled by UltraMicroPump and Micro4 (World Precision Instruments). The internal cannula was left in place for at least 1 min to prevent backflow. Electrophysiological recording or behavioral tests were begun 10 min later.

### Statistical Analysis

Data were analyzed by paired or unpaired *t-*tests, one-way ANOVA followed by Tukey’s test, or two-way ANOVA followed by the Bonferroni test, using GraphPad Prism 7.00 (GraphPad Software) or Origin 9.1 (OriginLab) software. Unless otherwise indicated, data are expressed as the mean ± SEM, and statistical significance was considered when *P <*0.05.

## Results

### CUMS Induces Depression-like Behaviors with vSub Hyperactivation in Mice

**Fig. 1:**
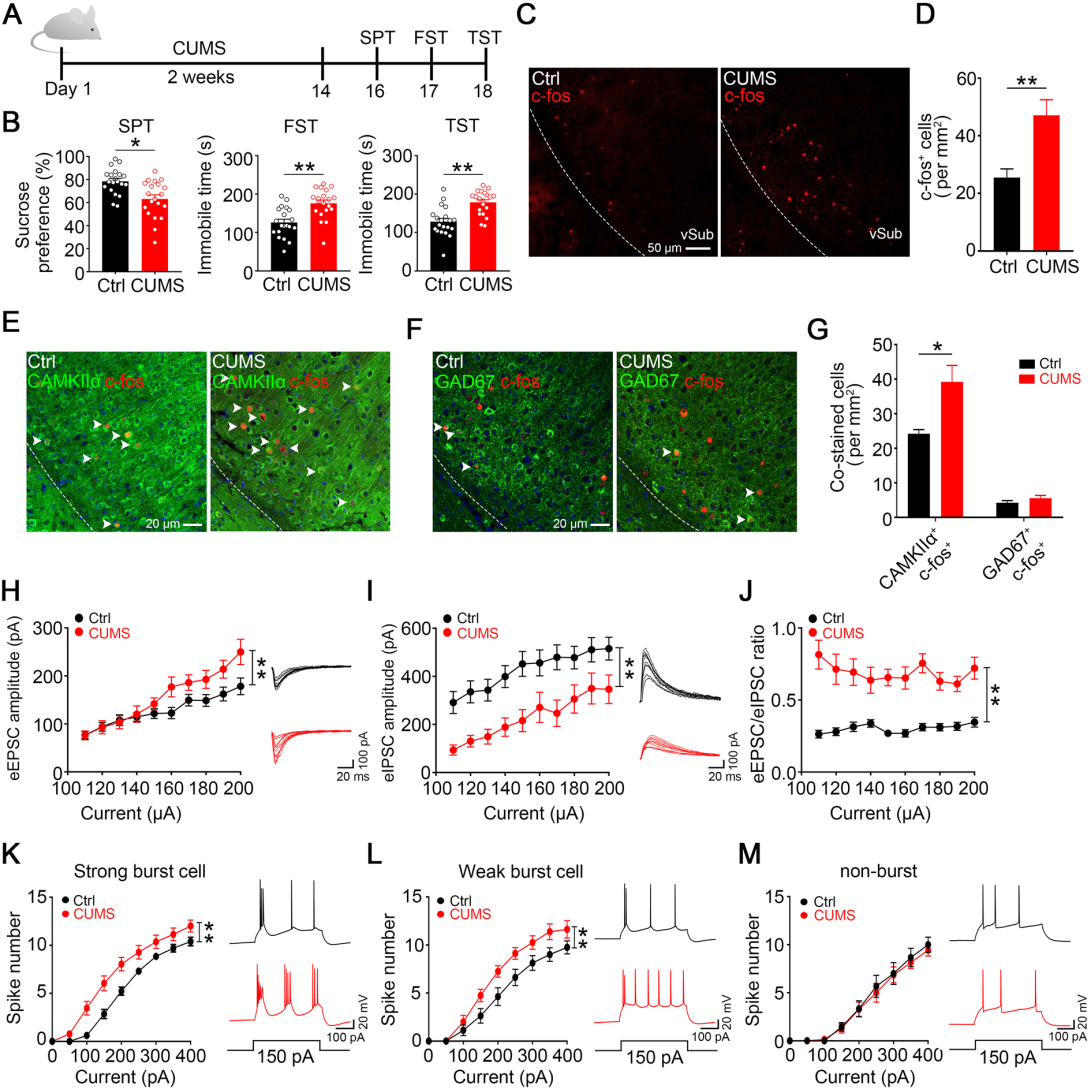
CUMS induces depression-like behaviors with glutamatergic hyperactivation and excitation/inhibition imbalance in the vSub. **A** Schematic of the CUMS procedures and behavioral tests. After 14 days of CUMS, mice are subjected to SPT, FST, and TST behavioral tests. **B** CUMS induces depression-like behaviors as shown by the decreased sucrose preference in the SPT (*P =* 0.0011, unpaired *t-*test, *n =* 20 mice per group) and the increased immobility time in the FST (*P =* 0.0002, unpaired *t-*test, *n =* 20 mice per group) and TST (*P <* 0.0001, unpaired *t-*test, *n =* 20 mice per group). **C, D** CUMS increases neural activity in the vSub, as shown by an increased number of c-fos-positive neurons measured by immunofluorescent staining (**C**) and quantitative analysis (**D**, *P =* 0.0059, unpaired *t-*test, *n* = 6 slices from 3 mice per group). **E–G** CUMS increases pyramidal activity in the vSub, as evidenced by an increase in c-fos-CaMKIIα but not c-fos-GAD67 co-labeled neurons. Fluorescent microscopic images of vSub section dual-immunostained for c-fos with CaMKIIα (**E**) or GAD67 (**F**) after CUMS (arrows indicate cells co-stained with c-fos-CaMKIIα and c-fos-GAD67 in **E** and **F**) and quantitative analysis (**G**) by the unpaired *t-*test (for c-fos-CaMKIIα co-labeled cells, *P =* 0.0119; for c-fos-GAD67 co-labeled cells, *P =* 0.2167; *n* = 6 slices from 3 mice per group). **(H–J)** CUMS induces excitation/inhibition imbalance in vSub pyramidal neurons. CUMS increases eEPSCs (**H**, *F* (1, 310) = 17.23, *P <*0.0001, two-way ANOVA, *n =* 13–22 cells from 5 mice per group) and decreases eIPSCs (**I**, *F* (1, 330) = 71.23, *P <*0.0001, two-way ANOVA, *n =* 13–22 cells from 5 mice per group), resulting in an increase in the eEPSC/eIPSC ratio (**J**, *F* (1, 310) = 200.3, *P <*0.0001, two-way ANOVA). Representative traces of eEPSCs and eIPSCs recorded by whole-cell patch-clamp in vSub brain slices at holding potentials of –70 and 0 mV, respectively are shown on the right in **H** and **I**. **K–M** CUMS increases the numbers of spikes in strong burst cells (**K**, *F* (1, 297) = 38.97, *P <*0.0001, two-way ANOVA, *n =* 13–22 cells from 5 mice per group) and in weak burst cells (**L**, *F* (1, 126) = 27.77, *P <*0.0001, two-way ANOVA, *n =* 8 cells from 5 mice per group), without influence on non-burst cells (**M**, *F* (1, 126) = 0.4311, *P =* 0.5126, two-way ANOVA, *n =* 8 cells from 5 mice per group), recorded by whole-cell patch-clamp in vSub brain slices. Representative traces in response to 150 pA are shown on the right. All data are expressed as the mean ± SEM, *P <0.05, **P <0.01.

To gain insight into the role of the vSub in stress-induced depression, the commonly used rodent depression CUMS model was used [28] (Fig. 1A), and no difference was found in water consumption, and locomotor activity after CUMS (data not shown). However, depression-like behaviors were induced, as evidenced by a significant difference in the sucrose preference test (SPT) and the elevated duration of immobility in the forced swimming (FST) and tail suspension (TST) tests (Fig. 1B). These results suggest the efficacy of the 14-day CUMS protocol to induce both anhedonia and hopelessness, the core symptoms of depression.

To investigate the involvement of the vSub in CUMS, we first applied c-fos immunostaining to vSub slices and found significantly increased c-fos-positive neurons after CUMS (Fig. 1C and D). Further c-fos co-staining with CaMKIIα (a marker of glutamatergic neurons in the hippocampus) and GAD67 (a marker of GABAergic neurons) demonstrated that most c-fos-positive cells were glutamatergic, and these c-fos and CaMKIIα co-labeled cells were significantly increased in the CUMS group (Fig. 1E–G). To further elucidate the mechanisms underlying the vSub hyperactivation, we recorded the local eEPSCs and eIPSCs in vSub pyramidal neurons to investigate the neural transmission. The increased glutamatergic and decreased GABAergic innervation of pyramidal neurons resulted in an elevated eEPSC/eIPSC ratio (Fig. 1H–J), suggesting a shift of excitatory and inhibitory balance (E/I balance) toward excitation in vSub after CUMS.

To measure the response of vSub pyramidal neurons to the current inputs, we recorded the action potential firing by whole-cell patch-clamp. The vSub pyramidal neurons have three subtypes based on their firing patterns elicited by current injection: strong burst*-*spiking initiated by low current injection (~50 pA) and high-frequency firing (~200 Hz, calculated by the interval between the first two action potentials), weak burst*-*spiking initiated by high current injection (~100 pA) and low-frequency firing (>50 Hz), and non-burst*-*spiking neurons characterized by lower-frequency firing (<50 Hz) [27, 29, 30]. The high-frequency bursts are crucial for the fidelity of neural transmission from the subiculum [27, 31]. We found that after CUMS treatment, the firing rates of both strong and weak burst neurons were increased in the vSub, but the non-burst neurons did not change (Fig. 1K–M). These data further confirm the hyperactivation of pyramidal neurons in the vSub, which might mediate the behavioral susceptibility to CUMS.

### The vSub Receives Dense MSDB Cholinergic Innervation Identified by Anterograde and Retrograde Tracing in Mice

**Fig. 2:**
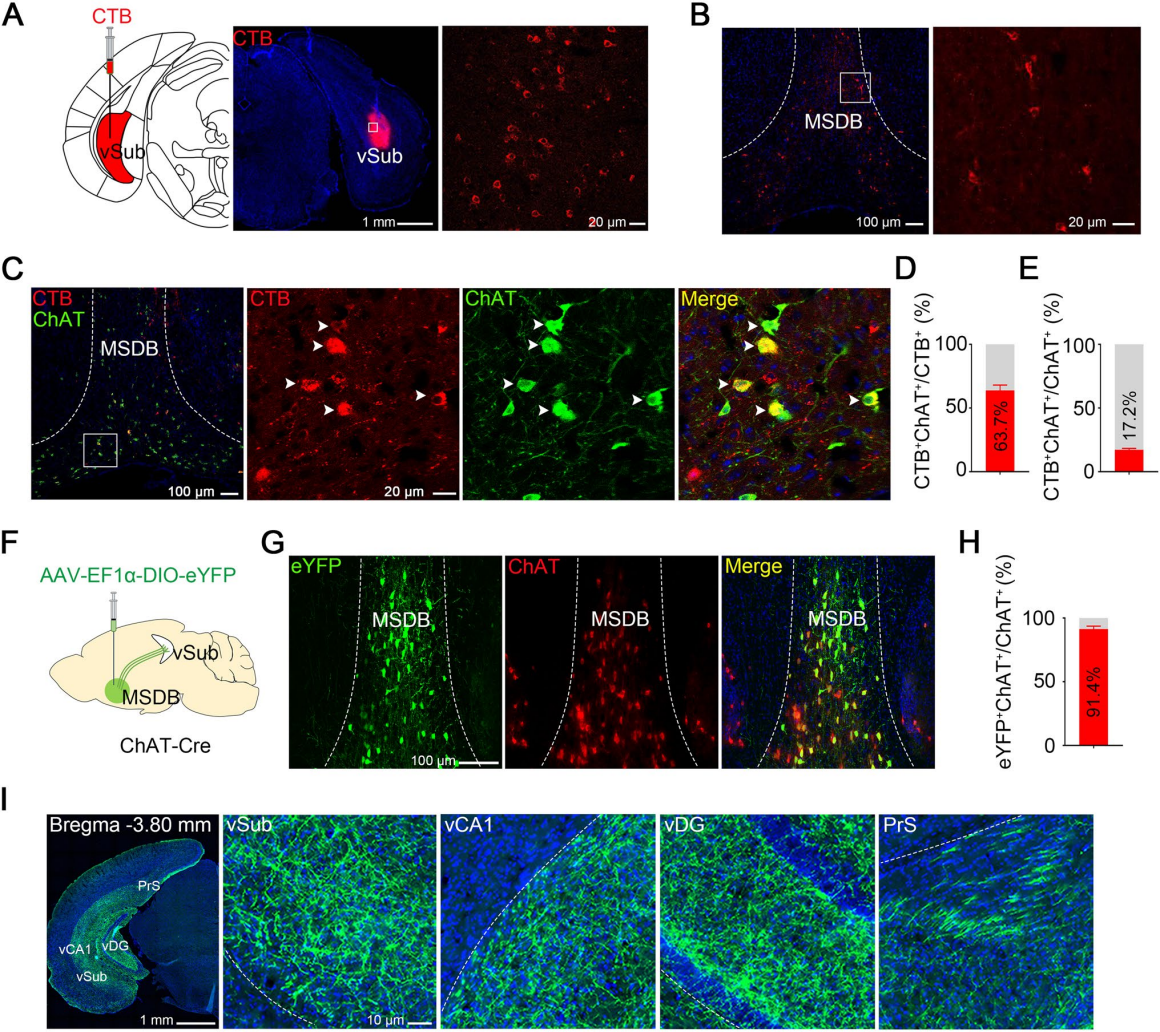
The vSub receives inputs from MSDB cholinergic neurons. **A** Schematic and representative images of CTB infusion into the vSub. **B** Representative images showing MSDB neurons retrogradely labeled with CTB projecting to the vSub. **C–E** MSDB neurons projecting to the vSub are mainly cholinergic. MSDB neurons retrogradely labeled with CTB co-stained with ChAT in coronal sections of the MSDB (**C**), quantification of co-labeled neurons showing that ~63% of CTB-positive neurons are cholinergic (**D**) and ~17% of the cholinergic neurons in the MSDB project to the vSub (**E**) (arrows indicate CTB-ChAT co-positive neurons; *n* = 6 slices from 3 mice). **F** Schematic of the MSDB infusion of AAV-EF1α-DIO-eYFP in ChAT*-*Cre mice. **G, H** Most cholinergic neurons in the MSDB are labeled with eYFP. Representative images showing ChAT and eYFP co-stained neurons in the MSDB (*n* = 6 slices from 3 mice). **I** The ventral hippocampus is densely innervated by MSDB cholinergic neurons. Representative images showing the distribution of cholinergic terminals (vSub, ventral subiculum; vCA1, ventral CA1; vDG, ventral dentate gyrus; PrS, presubiculum). All data are expressed as the mean ± SEM.

Clinical and preclinical studies have shown that ACh is increased in the brain during MDD [20, 21, 32]. MSDB cholinergic neurons are reportedly a key regulator of hippocampal excitability as the main source of cholinergic afferents to the hippocampus [33–35]. To identify MSDB neurons projecting to the vSub, we stereotaxically injected the retrograde tracer CTB into the vSub of C57 mice (Fig. 2A). After 10 days–12 days, large numbers of CTB-positive neurons were detected in the MSDB (Fig. 2B). Further immunofluorescent staining of choline acetyltransferase (ChAT), a marker of cholinergic neurons, revealed that ~63% of CTB-positive neurons were cholinergic, and ~17% of the cholinergic neurons in the MSDB were CTB-positive (Fig. 2C–E). To confirm direct innervation of the hippocampal subregions by MSDB cholinergic neurons, we applied anterograde tracing by stereotaxically infusing AAV-EF1α-DIO-eYFP into the MSDB of ChAT*-*Cre mice and measured the expression of eYFP after 28 days (Fig 2F and S2). eYFP was detected in ~90% of the cholinergic neurons in the MSDB (Fig. 2G and H), and dense eYFP-labeled terminals were observed in the ventral hippocampal subregions, including the vSub (Fig. 2I). These data together reveal a direct and dense vSub innervation by MSDB cholinergic neurons.

### Transient Restraint Stress Increases the ACh Signal in CUMS Mice

**Fig. 3:**
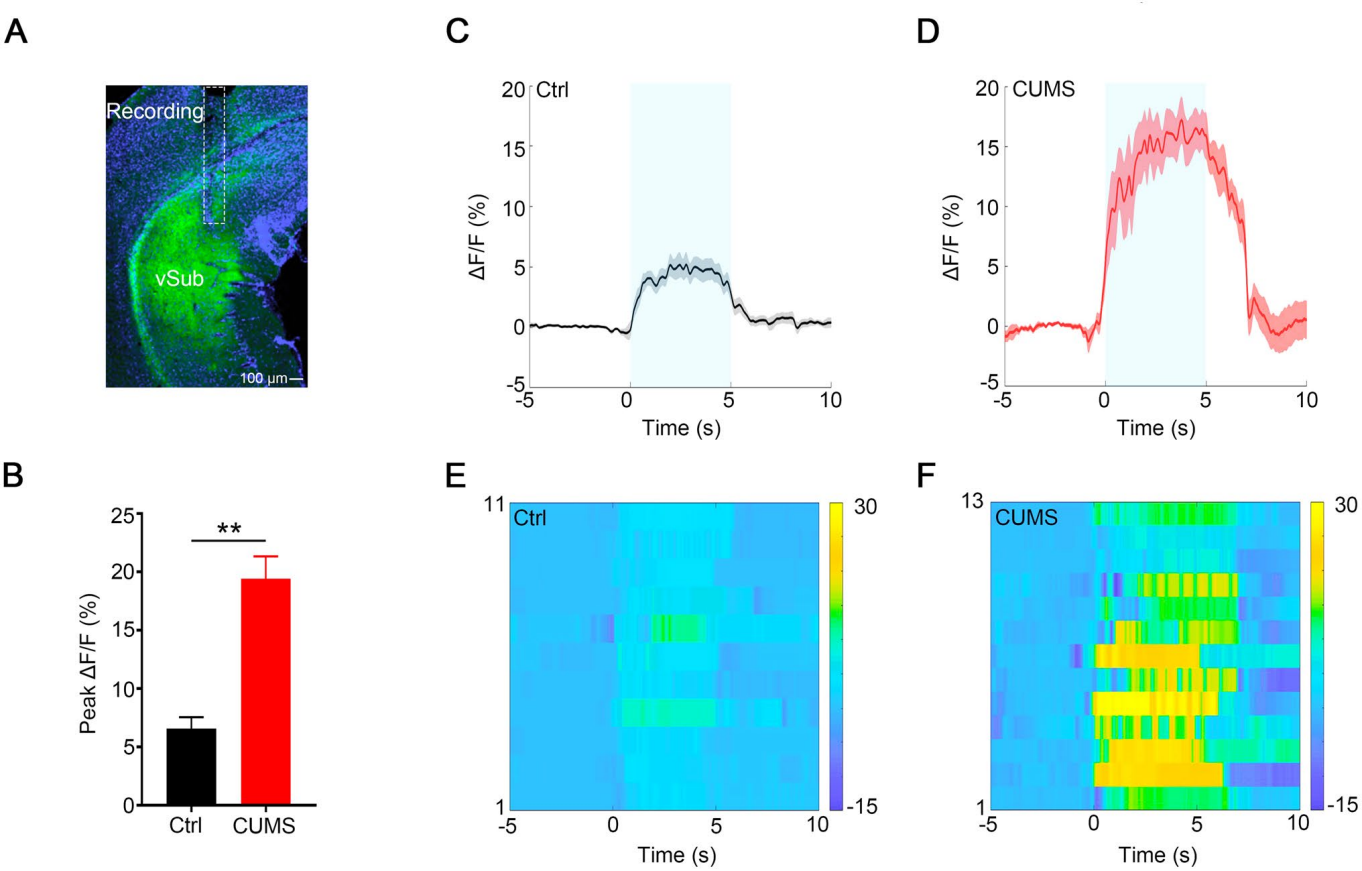
Transient restraint stress increases the ACh signal in CUMS mice. **A** Stereotaxic infusion of AAV-hSyn-GACh2.0 into the vSub of a C57 mouse. Representative image of GACh2.0 expression (green) and optical fiber (white rectangle) above the vSub (scale bar, 100 μm). **B** Quantification of peak fluorescence intensity in control and CUMS mice during 5-sec restraint stress (*P <*0.0001, paired *t-*test; *n* = 8 mice per group). **C, D** Average fluorescence transients in the vSub from control and CUMS mice during the behavioral transition. **E, F** Heat maps in the vSub of control and CUMS mice during the behavioral transition. All data are expressed as the mean ± SEM, **P < 0.01.

The MSDB has been implicated as a neural substrate of mood-related disorders [36, 37]. To explore the involvement of MSDB cholinergic activity in depression-like behaviors, we first determined the firing frequency of these neurons by *in vivo* electrophysiological recording during acute restraint and tail pinch, two of the stressors used in CUMS. These transient stresses increased the firing rates of MSDB cholinergic neurons (Fig. S1 A and B). To investigate the change of ACh level in the vSub after CUMS, we injected AAV-hSyn-GACh2.0 virus into the vSub to express the ACh sensor in neurons and then recorded the fluorescence signal during transient restraint for 5 s. The fluorescent ACh sensor GACh has been broadly applied for monitoring cholinergic transmission [38]. Compared to the control group, the fluorescence intensity in the CUMS group during transient restraint was increased (Fig. 3), indicating that CUMS increases the ACh level during stress, consistent with the increased firing rates of MSDB cholinergic neurons during stress as tested by *in vivo* electrophysiological recording.

### Stimulating the MSDB–vSub Cholinergic Pathway Induces vSub Pyramidal Hyperactivation along with Depression-like Phenotypes

**Fig. 4:**
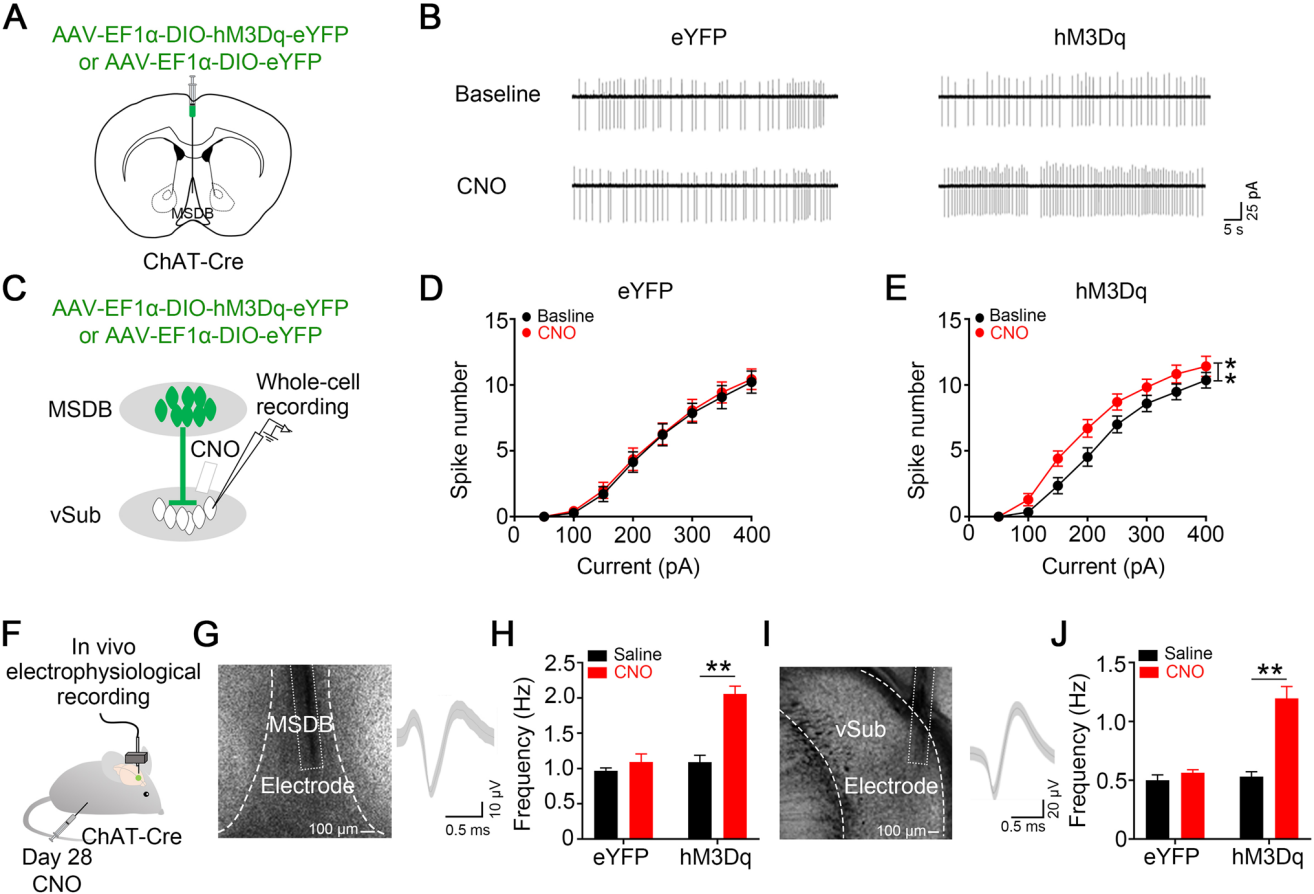
Chemogenetic stimulation of the MSDB-to-vSub cholinergic pathway induces hyperactivation of vSub pyramidal neurons. **A–E** Chemogenetic stimulation of MSDB cholinergic neurons increases the firing frequency of vSub pyramidal neurons recorded by *in vitro* electrophysiological recording. **A** Schematic of virus injection. AAV-EF1α-DIO-hM3Dq-eYFP (hM3Dq) is stereotaxically infused into the MSDB of ChAT*-*Cre mice (hM3Dq); AAV- EF1α-DIO-eYFP (eYFP) was used as control. After 28 days, brain slices containing the MSDB or vSub were prepared for recording. **B** Chemogenetic stimulation of MSDB cholinergic neurons by bath CNO in brain slices increases firing rates in the MSDB measured by cell-attached path-clamp. **C** Schematic of whole-cell patch-clamp recording from vSub pyramidal neurons in brain slices. **D, E** Chemogenetic stimulation of MSDB cholinergic terminals by bath CNO increases the firing rates of vSub pyramidal neurons (for eYFP groups, *F* (1, 234) = 0.2141, *P =* 0.644, two-way ANOVA, *n =* 14 cells from 5 mice per group; for hM3Dq groups, *F* (1, 270) = 22.64, *P <*0.0001, two-way ANOVA; *n =* 16 cells from 5 mice per group). **F–J** Chemogenetic stimulation of MSDB cholinergic neurons increases the vSub pyramidal firing frequency. **F** Schematic of recording from MSDB cholinergic or vSub pyramidal neurons. **G** Representative image of the recording electrode (white rectangle) in the MSDB (left) and typical superimposed average traces of putative cholinergic neurons (right). **H** Chemogenetic stimulation of MSDB cholinergic neurons by intraperitoneal (i.p.) injection of CNO increases their firing rates (for eYFP groups, *P =* 0.2472; for hM3Dq groups, *P =* 0.0002; *n* = 7–16 cells from 3–5 mice per group; paired *t-*test,). **I** Representative image of the recording electrode in the vSub (left) and typical superimposed average traces of a putative pyramidal neuron (right). **J** Chemogenetic stimulation of MSDB cholinergic neurons by i.p. injection of CNO increases the firing rates in vSub pyramidal neurons (for eYFP groups, *P =* 0.2766; for hM3Dq groups, *P <*0.0001; *n* = 16 cells from 6 mice per group; paired *t-*test). All data are expressed as the mean ± SEM, **P <0.01.

To investigate whether specifically activating the MSDB cholinergic neurons could activate vSub pyramidal neurons and induce depression-like behaviors, we applied the chemogenetic method by infusing AAV of Cre-dependent hM3Dq into the MSDB of ChAT*-*Cre mice (ChAT^cre^-hM3Dq mice) and then treated them with CNO (Fig. 4A). Chemogenetic stimulation of the MSDB cholinergic neurons was confirmed by cell-attached patch-clamp recording from eYFP-positive cells after bath application CNO to MSDB brain slices (Fig. 4A and B). The firing rates of pyramidal neurons in the vSub were increased after bath application of CNO to vSub brain slices from ChAT^cre^-hM3Dq mice, recorded by whole-cell patch-clamp of vSub pyramidal neurons, suggesting that stimulation of MSDB cholinergic neuron terminals in the vSub is enough to activate vSub pyramidal neurons (Fig. 4C–E). The firing rates of MSDB cholinergic neurons and vSub pyramidal neurons increased after i.p. injection CNO into ChAT^cre^-hM3Dq mice as confirmed by *in vivo* recording (Fig. 4F–J and S4).

**Fig. 5:**
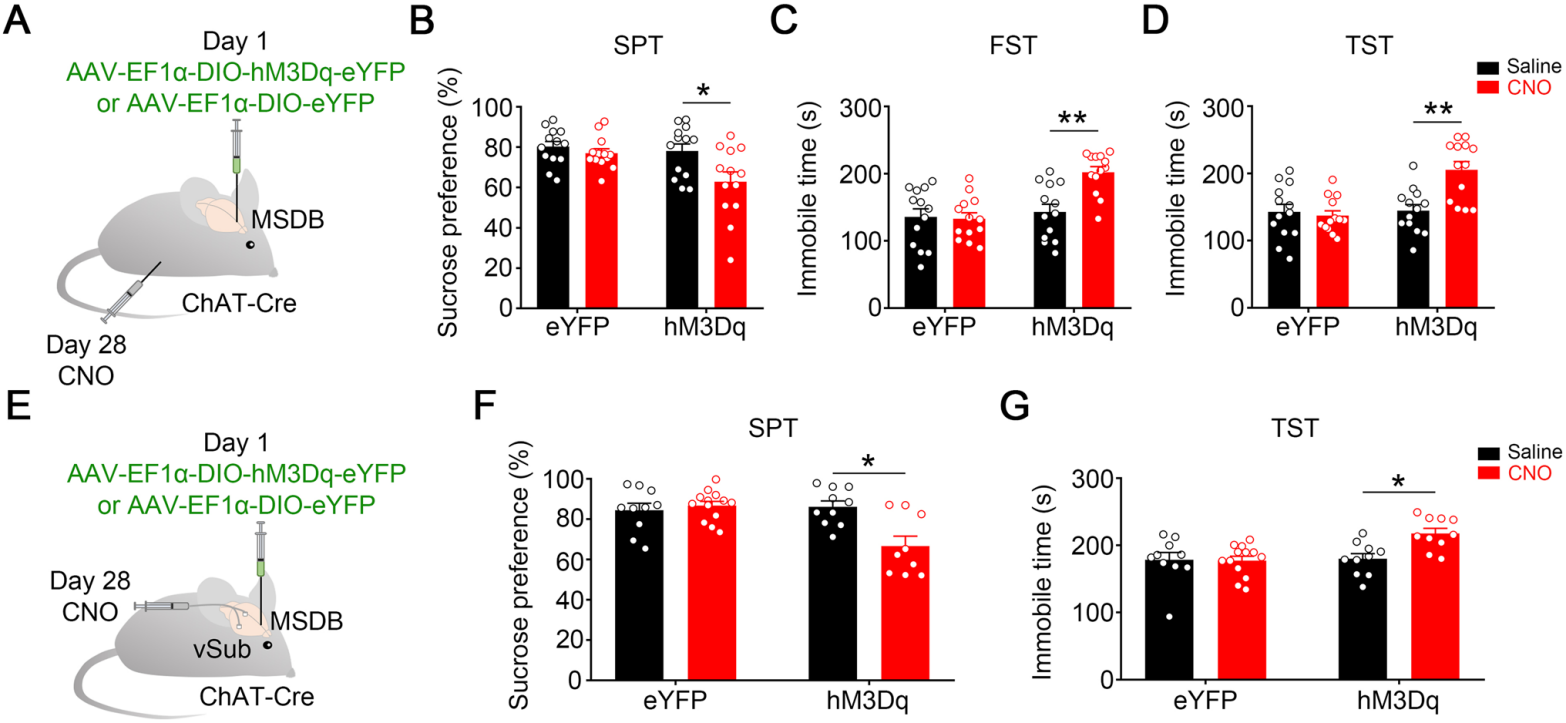
Chemogenetic stimulation of the MSDB-to-vSub pathway induces depression-like behaviors. **A** Schematic of virus injection and pharmacological procedure for behavioral tests. CNO is delivered by i.p. injection 28 days after virus injection. **B–D** Chemogenetic stimulation of MSDB cholinergic neurons in ChAT^cre^-hM3Dq mice by CNO i.p. injection induces depression-like behaviors evidenced by the decreased sucrose preference (**B**, for eYFP groups, *P =* 0.3399; for hM3Dq groups, *P =* 0.0189; *n* =13 mice per group; unpaired *t-*test) and increases the immobility time in the FST (**C**, for eYFP groups, *P =* 0.8628; for hM3Dq groups, *P =* 0.0004; *n* =13 mice per group; unpaired *t-*test) and TST (**D**, for eYFP groups, *P =* 0.6792; for hM3Dq groups, *P =* 0.0005; *n* =13 mice per group; unpaired *t-*test). **E** Schematic of virus injection and pharmacological procedure for behavioral tests. CNO is delivered by stereotaxic infusion into the vSub 28 days after virus injection. **F, G** Chemogenetic stimulation of the MSDB-vSub cholinergic pathway in ChAT^cre^-hM3Dq mice by bilateral infusion of CNO into the vSub induces depression-like behaviors evidenced by decreased sucrose preference (**F**, for eYFP groups, *P =* 0.5524; for hM3Dq groups, *P =* 0.0013; *n =* 10–13 mice per group; unpaired *t-*test), and increased immobility time in the TST (**G**, for eYFP groups, *P =* 0.9136; for hM3Dq groups, *P =* 0.0026; *n =* 10–13 mice per group; unpaired *t-*test). All data are expressed as the mean ± SEM, *P <0.05, **P <0.01.

Then we investigated whether hyperactivation of MSDB cholinergic neurons could induce depression-like behaviors in ChAT^cre^-hM3Dq mice. Compared to saline-injected controls, the ChAT^Cre^-hM3Dq mice injected with CNO i.p. showed a decreased sucrose preference, increased immobility duration in the FST and TST (Fig. 5A–D and S3), and decreased locomotor activity in the OFT (Fig. S5A), suggesting that activating MSDB induces depression-like behaviors. To confirm the role of the vSub in mediating the MSDB cholinergic activation to induce depression-like behaviors and exclude the possibility of an indirect role of a non-cholinergic MSDB projection, we infused CNO into the vSub of ChAT^cre^-hM3Dq mice through a cannula, and these mice also showed depression-like behaviors as tested by the SPT and TST (Fig. 5E–G) but had no effect on locomotor activity in the OFT (Fig. S5B).

Recent studies have shown co-transmission of ACh and GABA to the hippocampus in MSDB cholinergic neurons [39, 40], and we also found the co-release of GABA in the MSDB-vSub cholinergic pathway (Fig. S6 A–E). Next, we assessed whether activating GABA receptors in the vSub could affect depression-like behaviors. Bilaterally infusing muscimol (a GABA_A_ receptor agonist) into the vSub of wild-type mice (2 months old) did not induce depression-like behaviors, as measured by the SPT and TST (Fig. S6 F–H).

Taken together, these data demonstrated that stimulating MSDB-to-vSub cholinergic pathway induces depression-like behaviors.

### Atropine Reverses the Hyperactivation of vSub Pyramidal Neurons Induced by Activating the MSDB-vSub Cholinergic Pathway and Reduces Depression-like Behaviors

**Fig. 6:**
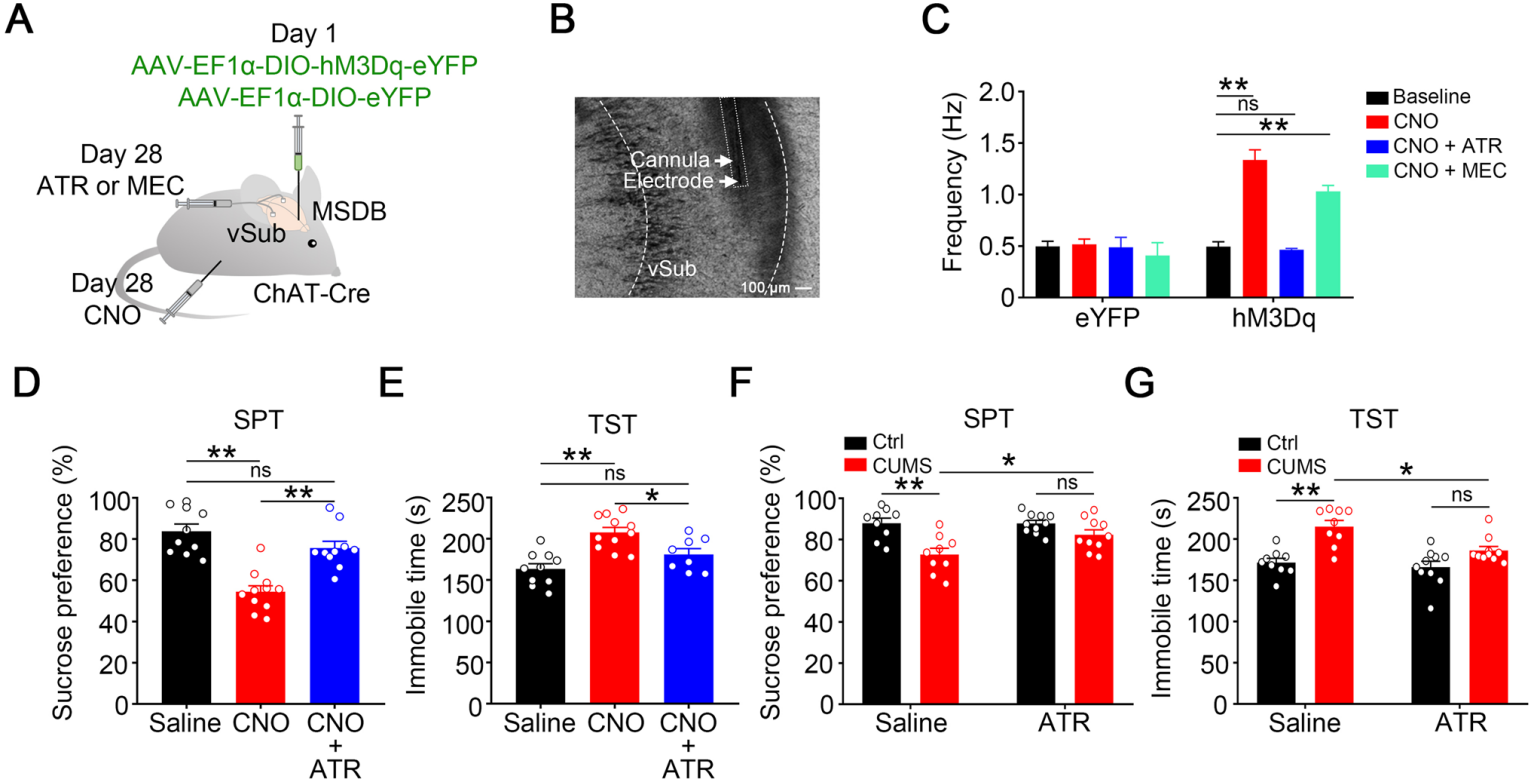
Atropine in the vSub reverses depression-like behaviors induced by activating the MSDB-to-vSub cholinergic inputs and CUMS. **A** Schematic of virus injection and pharmacological procedure. 28 days after AAV-EF1α-DIO-hM3Dq-eYFP or AAV-EF1α-DIO-eYFP is infused into the MSDB of ChAT*-*Cre mice, CNO is injected i.p., and 30 min later, atropine (ATR, a muscarinic cholinergic receptor antagonist) or mecamylamine (MEC, a nicotinic cholinergic receptor antagonist) is stereotaxically infused into the vSub on both sides, and the firing frequency of pyramidal neurons in the vSub is calculated from *in vivo* electrophysiological recordings. **B** Representative image of the cannula and recording electrode in the vSub. **C** Application of atropine but not mecamylamine reverses the effect of the chemogenetically-activated MSDB-vSub cholinergic pathway on vSub pyramidal neurons evidenced by the restored firing frequency (Two-way ANOVA, for hM3Dq groups, *F*(1, 54) = 28.34, *P <*0.0001; *post hoc* test: baseline *vs* CNO, *P <*0.0001; baseline *vs* CNO+ATR, *P* >0.9999; baseline *vs* CNO+MEC, *P =* 0.0009; CNO *vs* CNO+ATR, *P <*0.0001; CNO *vs* CNO+MEC, *P =* 0.0812; CNO+ATR *vs* CNO+MEC, *P =* 0.0025; *n =* 5–19 cells from 5 mice per group). **D, E** Application of atropine attenuates the depression-like behaviors induced by activation of the MSDB-vSub cholinergic pathway as evidenced by increased sucrose preference (**D**, one-way ANOVA, *F* (2, 28) = 22.74, *P <*0.0001; *post hoc* test: Saline *vs* CNO, *P <*0.0001; Saline *vs* CNO+ATR, *P =* 0.1987; CNO *vs* CNO+ATR, *P =* 0.0002; *n =* 8–11 mice per group) and decreased immobility time in the TST (**E**, one-way ANOVA, *F* (2, 26) = 12.88, *P =* 0.0001; *post hoc* test: Saline *vs* CNO, *P <*0.0001; Saline *vs* CNO+ATR, *P =* 0.1785; CNO *vs* CNO+ATR, *P =* 0.0218; *n =* 8–11 mice per group). **F, G** Application of atropine reverses CUMS-induced depression-like behaviors as evidenced by sucrose preference (**F**, two-way ANOVA, *F* (1, 34) = 18.49, *P =* 0.0001, *post hoc* test: Ctrl + Saline *vs* CUMS + Saline, *P =* 0.0007; CUMS + Saline *vs* CUMS + ATR, *P* =0.0475; Ctrl + ATR *vs* CUMS + ATR, *P =* 0.4865; *n =* 9–10 mice per group) and TST (G, two-way ANOVA, *F* (1, 34) = 25.99, *P <*0.0001, *post hoc* test: Ctrl + Saline *vs* CUMS + Saline, *P =* 0.0002; CUMS + Saline *vs* CUMS+ATR, *P* = 0.0125; Ctrl + ATR *vs* CUMS + ATR, *P =* 0.1089; *n =* 9–10 mice per group).

According to the literature, both muscarinic and nicotinic ACh receptors are involved in depression [41, 42]. To explore which cholinergic receptor mediates the MSDB-to-vSub cholinergic pathway, we injected the muscarinic receptor antagonist atropine or the nicotinic receptor antagonist mecamylamine into the vSub of ChAT^Cre^-hM3Dq mice. Thirty minutes after CNO injection i.p., we found that the activation of vSub pyramidal neurons was eliminated by bilateral infusion of atropine but not mecamylamine into the vSub (Fig. 6A–C). Simultaneously, the vSub infusion of atropine attenuated the CNO-induced depression-like behaviors in ChAT^Cre^-hM3Dq mice (Fig. 6D and E).

Based on evidence that muscarinic receptor antagonists play a role in activating the MSDB-vSub cholinergic pathway in depression-like behaviors, we next tested the antidepressant effects of atropine in the vSub on CUMS mice. Five days after bilateral delivery of atropine into the vSub, we found an increased sucrose preference and decreased immobility time in CUMS mice (Fig. 6F and G). Together, these data indicate that muscarinic ACh receptor mediates the activation of the MSDB-to-vSub cholinergic pathway to induce depression-like behaviors.

## Discussion

**Fig. 7:**
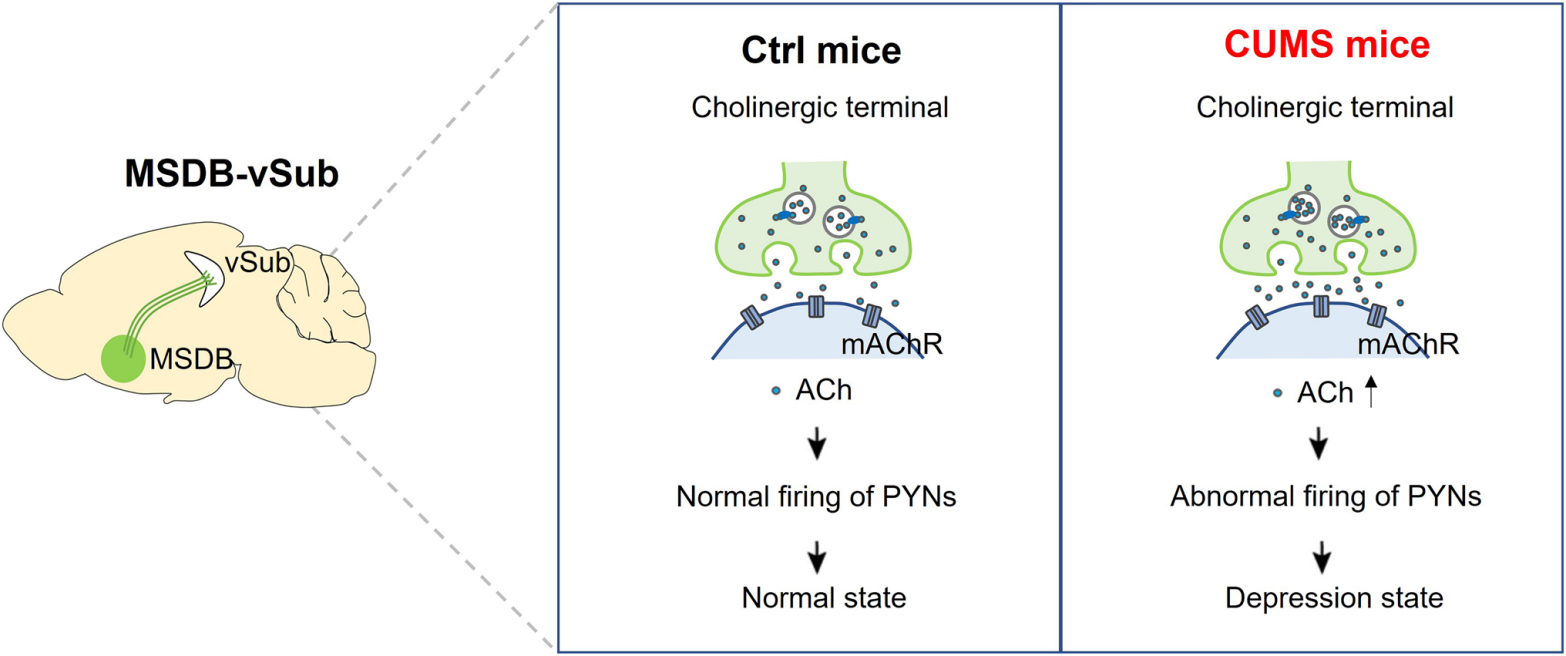
Schematic of the role of the MSDB-vSub cholinergic pathway in depression. The vSub is densely innervated by the MSDB. Excessive acetylcholine release in the MSDB-vSub pathway induces hyperactivation of vSub pyramidal neurons (PYNs) and depression-like behaviors *via* muscarinic receptors (mAChR).

The present study establishes that the MSDB-vSub cholinergic pathway is critical in depression-like behaviors. Accompanied by CUMS-induced depression-like behaviors, vSub pyramidal neurons were hyperactivated, as revealed by a shift of local transmission toward excitation, and increased burst-firing rates. Next, we showed a dense MSDB cholinergic innervation in the vSub, and the level of ACh in the vSub was significantly increased by transient stress in the CUMS mouse model. Chemogenetically stimulating the MSDB-vSub cholinergic pathway hyperactivated vSub pyramidal neurons and induced depression-like behaviors, and atropine reverse these effects in the vSub, suggesting a causal effect of hyperactivity of MSDB-vSub cholinergic transmission on depression-like behaviors (Fig. 7). To the best of our knowledge, this is the first report revealing the critical role of the MSDB-vSub cholinergic pathway in depression, providing novel targets for clinical intervention.

Brain abnormalities in patients with depression have been reported using a combination of functional brain imaging approaches. The major functional networks include the default mode network (DMN), responsible for resting-state introspection and rumination; the salience network (SAL), which processes salient information from external sources; and the central executive network (CEN), responsible for working memory and attention [43]. The DMN is composed of connections between the dorsal lateral prefrontal cortex, cingulate cortex, amygdala, and hippocampus [44]. Studies have demonstrated an increase in the activity of the DMN in depressed patients and decreases in the SAL and CEN, consistent with increased rumination and introspection and decreased association with external inputs in these patients [45, 46]. As the main subregion controlling the output of the hippocampus, the subiculum projects to the prefrontal cortex, nucleus accumbens, amygdala, and septal nuclei [47, 48], regions involved in depressive phenotypes. Although clinical studies have reported a significantly decreased volume of the subiculum in depressed patients [49], how subiculum dysfunction contributes to depression remains elusive. In the present study, we found that vSub pyramidal neurons were hyperactivated during depression, consistent with the increased DMN activity and rumination in patients [46]. Hyperactivation of glutamatergic vSub neurons can also cause neuron death, which may underlie the vSub atrophy [50]. These studies strongly suggest that hyperactivation of the vSub plays a crucial role in the pathogenesis of depression. Consistent with previous findings [30, 51], our experiments showed that chronic stress can induce depression-like behaviors and enhance neuronal activity in the vSub.

Then we studied the circuit mechanisms that may lead to vSub hyperactivation. Using tracing methods, we identified that the vSub receives densely cholinergic innervation from the MSDB, a region enriched in cholinergic neurons [52]. Consistent with the reports that cholinergic transmission is significantly increased in MDD patients and animal models [18, 20, 21, 53, 54], we found that selectively activating this circuit induce vSub hyperactivation along with depression-like behaviors, and these effects were blocked by the muscarinic ACh receptor antagonist atropine in the vSub. Considering that vSub pyramidal neurons are also hyperactivated in the CUMS model, and muscarinic receptor type-1 (M1) and muscarinic receptor type-3 (M3) are enriched in pyramidal neurons [55], our results suggest that hyperactivation of MSDB cholinergic neurons induces depression by activating vSub pyramidal neurons through muscarinic ACh receptors.

Extensive evidence has shown that muscarinic ACh receptor antagonists such as scopolamine exert fast*-*onset and sustained antidepressant effects in MDD patients and animals [23, 56–58]. The nicotinic antagonist mecamylamine has also been reported to have antidepressant*-*like activity in rodents, including reduced immobility in the FST and increased sucrose preference [59, 60]. With our findings, inhibitors of muscarinic but not nicotinic ACh receptors had antidepressant effects, indicating that these effects depend on the brain nuclei or receptor subtypes involved. In the future, more work is needed to determine the potential role of subtypes of muscarinic ACh receptors in depression.

The basal forebrain cholinergic circuit plays an essential role in memory, cognitive flexibility, navigation, sleep, and appetite control [61–63], all of which can be dysregulated in patients suffering from depression [64, 65]. The neuron in MSDB has three types: cholinergic, GABAergic, and glutamatergic. Many cholinergic neurons are also GABAergic neurons. GABAergic neurons act as theta pacemakers for the hippocampus *via* the innervation of hippocampal GABAergic interneurons. Although hippocampal theta oscillation underlies learning, memory, and navigation behaviors in animals, we did not observe any depression-like behavior when applying a GABA agonist. Dysregulation of valence processing is the core feature of depression, and the medial septum is critical for valence processing through the glutamatergic projection to the hippocampus [66]. It is still controversial whether septo-hippocampal neurons contribute to theta oscillation in the hippocampus [67]. Interestingly, it has been reported that activation of these neurons suppresses hippocampal ripple oscillations through muscarinic receptors [67], and ripple oscillation is regarded as a cognitive biomarker for learning, memory, and motion planning [68, 69]. Here we found that the cholinergic neurons in the MSDB are also critical for depression-like behaviors; their activation might also contribute to the cognitive deficits in depressed patients through dysregulation of oscillation in the hippocampus.

In summary, our findings elucidate the pathophysiological mechanisms of the MSDB-vSub cholinergic pathway in depression; this provides insight into a new strategy to treat MDD through modulating ACh neurotransmitters.

## Supplementary Information

Below is the link to the electronic supplementary material.

Supplementary file1 (PDF 734 KB)
