# Supplementary material for: Basal Forebrain Cholinergic Innervation Induces Depression-Like Behaviors Through Ventral Subiculum Hyperactivation
Source: Neurosci Bull. 2022 Nov 7;39(4):617–30. doi: 10.1007/s12264-022-00962-2 (PMC10073402; doi:10.1007/s12264-022-00962-2)
Supplement: Supplementary file 1 — Supplementary file1 (PDF 734 KB) [file 12264_2022_962_MOESM1_ESM.pdf]

Supplementary Materials

Supplementary Figures

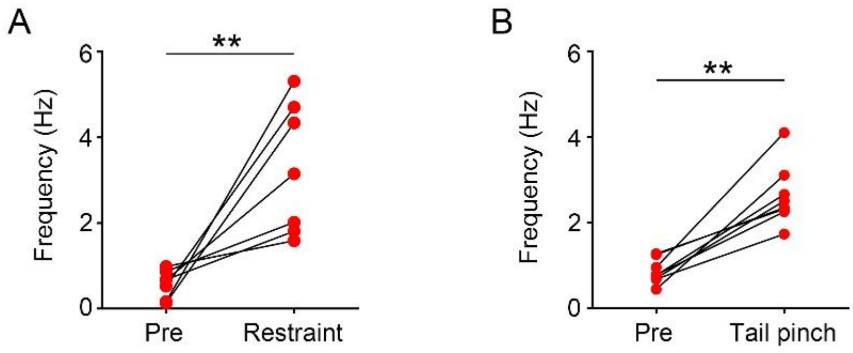

Fig. S1 Acute stress increases cholinergic activity in the MSDB. A, B Significantly increased spontaneous firing rates of MSDB cholinergic neurons in the stress models of acute restraint (**A**, paired *t*-test, $P = 0.0077$; $n = 7$ cells from 5 mice) and tail pinch (**B**, paired *t*-test, $P = 0.0004$; $n = 8$ cells from 6 mice).

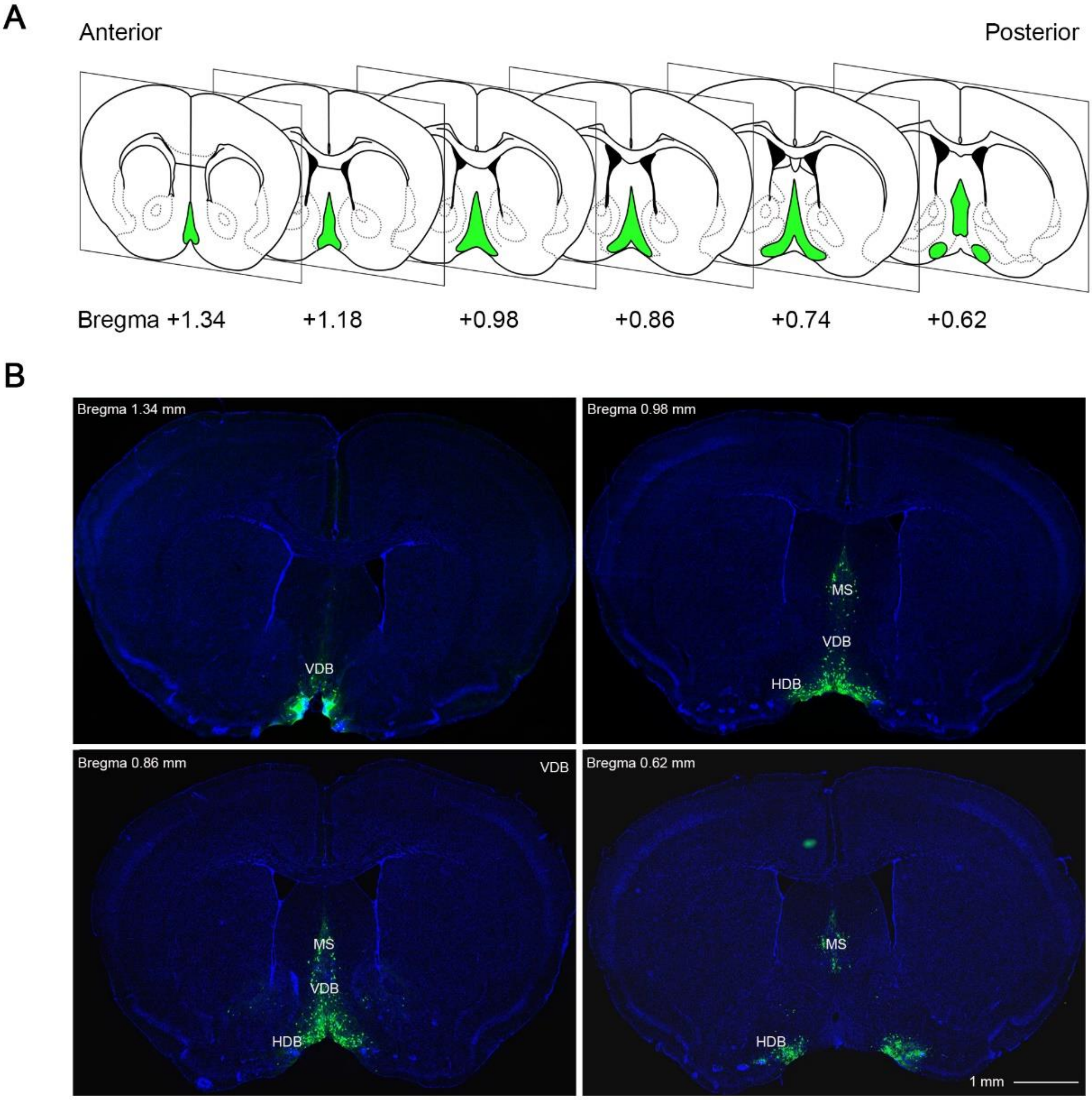

Fig. S2 Confirmation of entire MSDB infection by viral injection. A Coronal sections of MSDB, consisting of the medical septum (MS), vertical diagonal band of Broca (VDB), and horizontal diagonal band of Broca (HDB). **B** Representative images showing that injections of AAV-EF1α-DIO-eYFP into the MSDB in ChAT-Cre mice are sufficient to target the full extent of the MSDB. Scale bar, 1 mm.

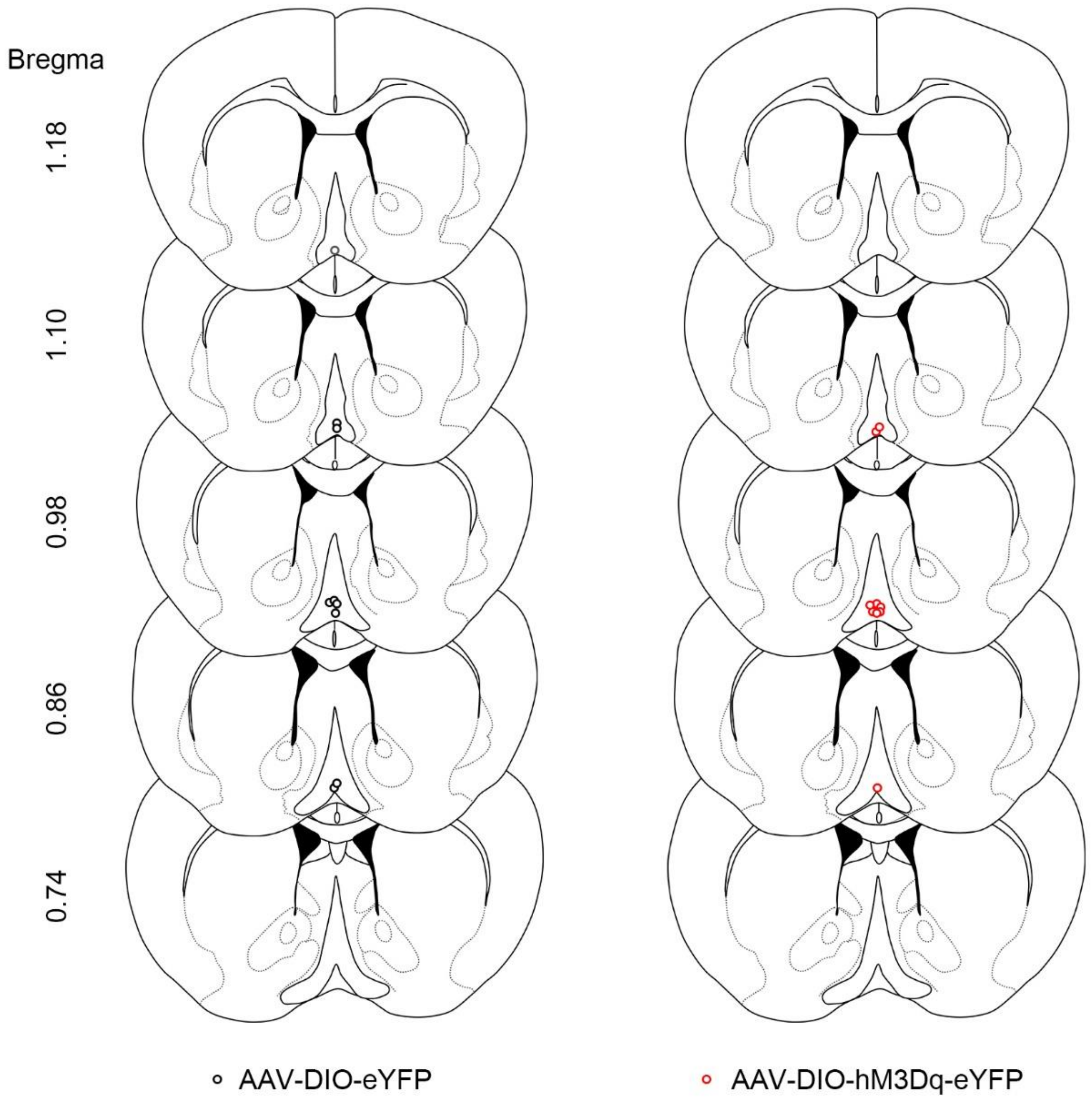

Fig. S3 Verification of injection sites in the MSDB. Anterior to posterior brain slices from ChAT-Cre mice following AAV-EF1α-DIO-eYFP virus injection into the MSDB. Black (eYFP group) and red (hM3Dq group) circles indicate the location of injection sites in the MSDB.

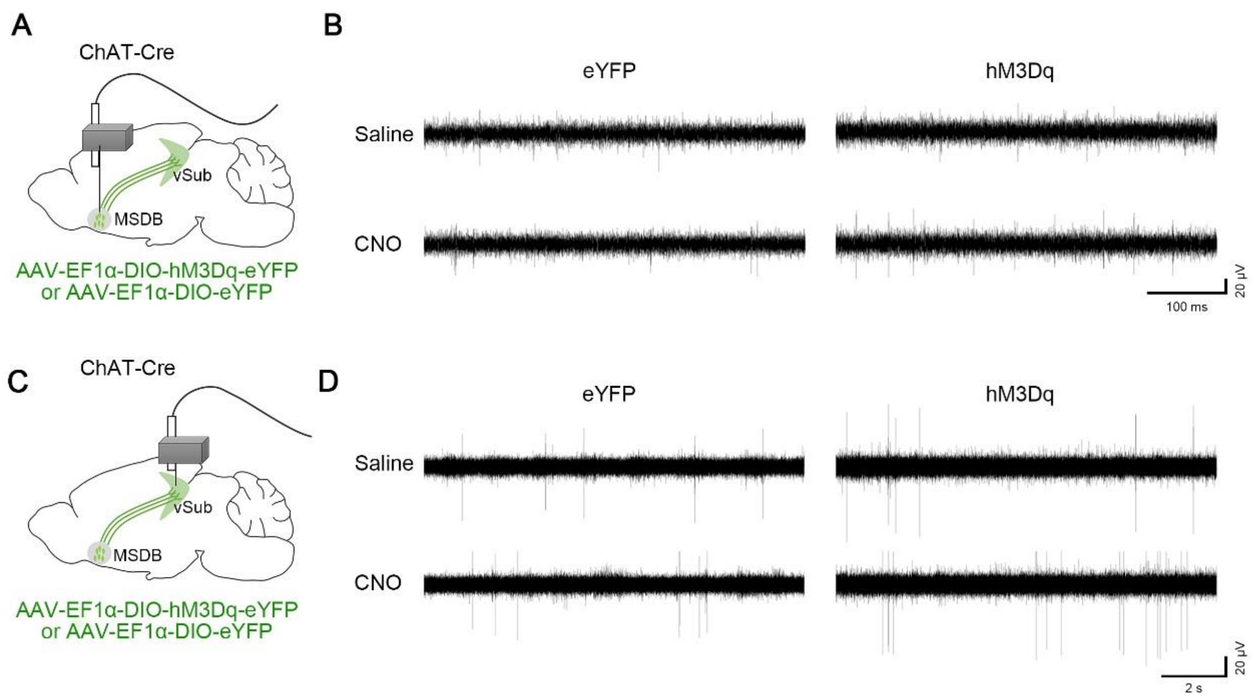

Fig. S4 Sample traces of in *vivo* electrophysiological recording**. A, C** Schematic of *in vivo* electrophysiological recording from MSDB cholinergic neurons (**A**) and vSub pyramidal neurons (**C**) after chemogenetic virus injection into the MSDB of ChAT-Cre mice. **B, D** Sample traces of recordings in the MSDB (**B**) and vSub (**D**) for figure 4H and 4J.

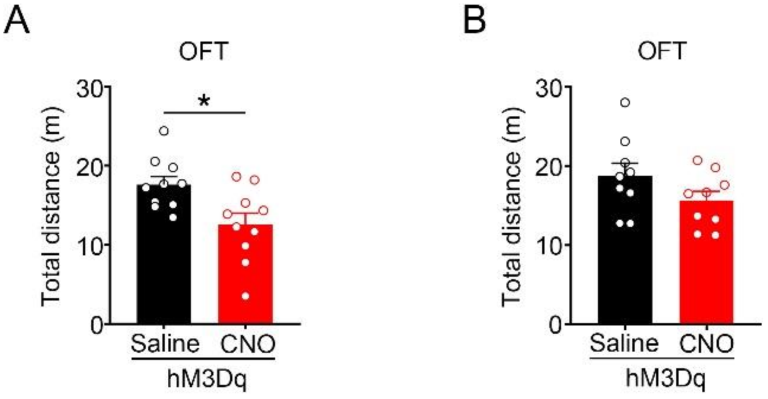

Fig. S5 Locomotor activity under CNO treatment in ChAT^{Cre}-hM3Dq mice. A Chemogenetic stimulation of MSDB cholinergic neurons in ChAT^{cre}-hM3Dq mice by i.p. injection of CNO decreases locomotor activity measured by the average total distance traveled in the open field test (OFT, unpaired *t*-test, $P = 0.0113$, $n = 10$ mice per group). **B** Chemogenetic stimulation of the MSDB-vSub cholinergic pathway in ChAT^{cre}-hM3Dq mice by local application of CNO into the vSub through a cannula does not change the locomotor activity as measured by the average total distance traveled in the OFT (unpaired *t*-test, $P = 0.1383$, $n = 9$ mice per group).

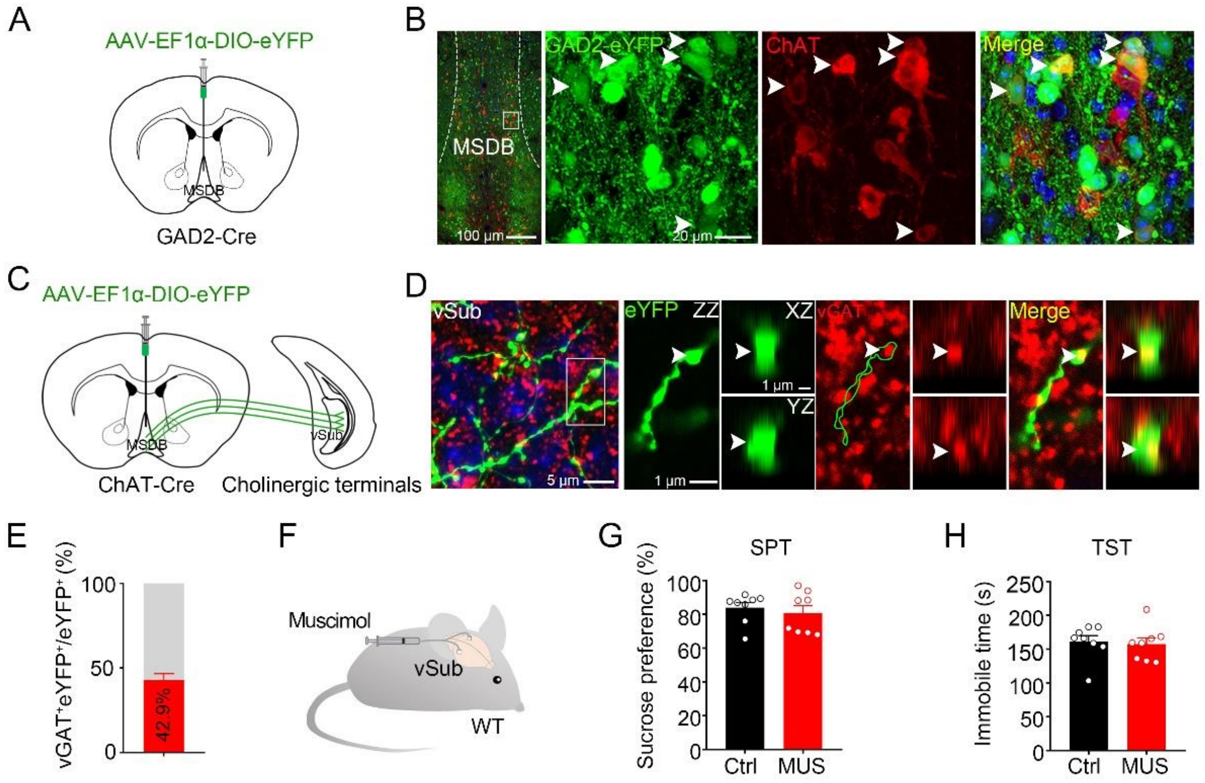

Fig. S6 Neurons in the MSDB-vSub cholinergic pathway co-release GABA but GABA agonist application into the vSub does not affect the depression-like behaviors. A, B MSDB cholinergic neurons express GAD2 (a GABAergic neuron marker). MSDB infusion with AAV-EF1α-DIO-eYFP in GAD2-Cre mice to label GAD2-positive neurons (**A**), and an MSDB slice immuno-stained with ChAT (**B**) (arrows, eYFP and ChAT co-positive neurons). **C–E** MSDB cholinergic terminals in vSub express vGAT (vesicular GABA transporter). Representative images after AAV-EF1α-DIO-eYFP infusion into the MSDB of ChAT-Cre mice to label cholinergic neurons (**C**), vSub brain slices after immuno-staining with vGAT (**D**) (arrowheads, eYFP and vGAT co-positive terminals), and quantitative analysis (**E**, $n = 6$ slices from 3 mice). **F–H** Muscimol in vSub does not affect depression-like behaviors. **F** Schematic of muscimol (MUS, a $GABA_A$ receptor agonist) infusion in wide-type mice. vSub infusion of muscimol in wild-type mice does not induce depression-like behaviors as measured by the SPT (**G**, unpaired *t*-test, $P = 0.5645$, $n = 8$ mice per group) and TST (**H**, unpaired *t*-test, $P = 0.7856$, $n = 8$ mice per group).
